# Evaluation of Comprehensive Gene Expression and NK Cell-Mediated Killing in Glioblastoma Cell Line-Derived Spheroids

**DOI:** 10.3390/cancers13194896

**Published:** 2021-09-29

**Authors:** Takayuki Morimoto, Tsutomu Nakazawa, Ryosuke Matsuda, Fumihiko Nishimura, Mitsutoshi Nakamura, Shuichi Yamada, Ichiro Nakagawa, Young-Soo Park, Takahiro Tsujimura, Hiroyuki Nakase

**Affiliations:** 1 Department of Neurosurgery, Nara Medical University, Kashihara, Nara 634-8521, Japan; t.morimoto@naramed-u.ac.jp (T.M.); rmatsuda@naramed-u.ac.jp (R.M.); fnishi@naramed-u.ac.jp (F.N.); mnaka@grandsoul.co.jp (M.N.); syamada@naramed-u.ac.jp (S.Y.); nakagawa@naramed-u.ac.jp (I.N.); park-y-s@naramed-u.ac.jp (Y.-S.P.); nakasehi@naramed-u.ac.jp (H.N.); 2 Grandsoul Research Institute for Immunology, Inc., Uda, Nara 633-2221, Japan; 3 Clinic Grandsoul Nara, Uda, Nara 633-2221, Japan; takahiro@grandsoul.co.jp

**Keywords:** glioblastoma, NK cell, spheroid model

## Abstract

**Simple Summary:**

Glioblastoma (GBM) is the most aggressive primary malignant brain tumor in adults. Despite standard treatment, including surgery, chemotherapy, and radiotherapy, it is associated with poor survival. Immunotherapy is a promising alternative for patients with GBM. Natural killer (NK) cells are possible promising targets in GBM treatment because of their potent cytotoxic effect. We previously reported that highly activated and ex vivo-expanded NK cells, or genuine induced NK cells (GiNK), exert a greatly cytotoxic effect on GBM cells. In this study, we investigated the potential of NK cell-based immunotherapy for GBM, which we evaluated using an ex vivo three-dimensional GBM cell-derived spheroid model. Our results indicated that the NK cells had an anti-tumor effect on the spheroid models. Our findings could lead to the development of future NK cell-based immunotherapies for GBM.

**Abstract:**

Glioblastoma (GBM) is the most common and aggressive primary brain tumor, with a dismal prognosis. Natural killer (NK) cells are large granular lymphocytes with natural cytotoxicity against tumor cells, and they should be established for the novel treatment of patients with GBM. We previously reported highly activated, and ex vivo-expanded NK cells derived from human peripheral blood, designated genuine induced NK cells (GiNK), which were induced by specific culture conditions and which exerted a cytotoxic effect on GBM cells via apoptosis. Here, we comprehensively summarize the molecular characteristics, especially focusing on the expression of stem cell markers, extracellular matrix markers, chemokines, chemokine receptors, and NK receptor ligands of spheroids derived from GBM cell lines as compared with that of two-dimensional (2D) adherent GBM cells via microarray. The spheroid had upregulated gene expression of stem cell markers, extracellular matrix markers, chemokines, chemokine receptors, and NK cell inhibitory receptor ligands compared with the 2D adherent GBM cells. Preclinical evaluation of the NK cells was performed via an ex vivo 3D spheroid model derived from GBM cell lines. In the model, the NK cells accumulated and infiltrated around the spheroids and induced GBM cell death. Flow cytometry-based apoptosis detection clearly showed that the NK cells induced GBM cell death via apoptosis. Our findings could provide pivotal information for NK cell-based immunotherapy for patients with GBM.

## 1. Introduction

Glioblastoma (GBM) is the most common and lethal primary brain tumor, classified as grade IV by the World Health Organization (WHO) [1]. The median overall survival (mOS) does not exceed 15 months after standard-of-care treatment, which consists of maximal surgical resection followed by radiotherapy and adjuvant temozolomide [2]. New strategies are needed for treating patients with GBM, and immunotherapy can be a promising adjuvant treatment. Several such clinical trials have recently been reported [3,4,5,6], but these therapies did not improve the OS in patients with GBM.

Natural killer (NK) cells were discovered more than 40 years ago and are considered to play an important role in controlling virus infections and tumor progression to mediate cytotoxicity and produce cytokines [7,8,9]. NK cell development and maturation are dependent on type I interferon (IFN) and the cytokines interleukin (IL)-12, IL-15, and IL-18 [10,11,12,13] and are trained through signals transduced by the activation and inhibitory receptors [14,15,16,17].

While tumor-associated macrophages (TAMs) are correlated with the clinical prognosis and grade of gliomas [18], the proportion of infiltrating cytotoxic NK cells is related with good prognosis [19]. However, in the brain tumor microenvironment (TME) of GBM, the lymphocyte compartment is mostly composed of T cells, with fewer NK cells and B cells [20]. Furthermore, immunosuppression is induced, causing tumor-infiltrating lymphocyte (TIL) anergy, recruiting regulatory T cells (Treg) and activating immune checkpoints [21,22,23,24,25,26]. Adoptive immunotherapy, including NK cell-based immunotherapy, aims to reach and activate the immune cells.

We previously reported genuine induced NK cells (GiNK), highly purified and expanded human NK cells derived from peripheral blood mononuclear cells (PBMCs) using a chemically defined and feeder-free method such as cancer cells and that exhibited high NK activity for GBM cells cultured in a standard two-dimensional (2D) culture system [27]. The 2D monolayer cell lines grown in serum-containing medium are one of the most important and common tools for studying GBM in vitro. This approach allows better understanding of GBM cell biology by simplifying studies and sidestepping ethical concerns. However, reflecting the therapeutic effect on patients with GBM is difficult because 2D culture cannot fully reproduce the brain TME of GBM where immunosuppression is induced. Therefore, it is necessary to construct an environment as similar as possible to the GBM microenvironment [28]. Xenograft models using severely immunodeficient mice such as NOD/Shi-scid IL-2RɤKO (NOG) mice have also long been used to study tumor development upon the engraftment of human cells into immunodeficient mice [29,30]. On the other hand, 3D culture of GBM cells reproduces the spatial organization and TME factors of in vivo micro-tumors more accurately [31]. Nevertheless, the anti-tumor effect of NK cells has never been reported in this model.

The 3D models are more suitable for representing an anti-cancer therapeutics resistance profile than 2D cell culture models because of the inherent metabolic gradients resulting in the multilayer structure [32], enhanced production and deposition of tumor extracellular matrix (ECM) proteins [33], and enriched glioma stem cell (GSC) markers [34]. Various chemokines and their receptors play important roles in GBM formation, progression, and therapeutics resistance [35]. Based on the anti-cancer therapeutics resistance profile of the 3D spheroid model, the expression of chemokines and their receptors should be investigated. However, few studies have comprehensively investigated the molecular characteristics of spheroids derived from GBM cell lines. Further, the anti-tumor effect of NK cells on GBM cell line-derived spheroids (GBM-sp) has not been elucidated. Despite the appealing aspect of NK cells in tumor immunity, there have been few investigations of the anti-tumor effect of NK cells against GBM cells using models other than the 2D monolayer-culture model.

The present study was specifically aimed at investigating the anti-tumor effect of primary human NK cells expanded from our specific culture conditions on a GBM cell-derived 3D spheroid model. We also used microarray to analyze the molecular characteristics of the GBM-sp, specifically focusing on the expression of stem cell markers, ECM markers, chemokines, chemokine receptors, and NK receptor ligands, and compared them with those of a 2D culture model. Our findings could provide pivotal information for clinical trials of NK cell-based immunotherapy in patients with GBM.

## 2. Materials and Method

### 2.1. Gene Expression Based on The Cancer Genome Atlas (TCGA) Data Set

To detect the expression patterns of the representative stem cell markers (Nestin, SOX2, STAT3, CD133), ECM markers (FN1, COL1A1, COL6A1, LTBP1), chemokines (CXCL12, CXCL16, CX3CL1, CCL2), and chemokine receptors (CXCR1, CXCR2, CXCR4, CCR5) in gliomas, we examined the RNA sequencing data of gliomas from TCGA database in the GlioVis data portal for the visualization and analysis of brain tumor expression data sets [36].

### 2.2. Cell Lines

We used four standard, human GBM cell lines: T98G, U87MG, LN-18 (American Type Culture Collection, Manassas, VA, USA), and U251MG (Japanese Collection of Research Bioresources Cell Bank, Osaka, Japan). We maintained the cells in Dulbecco’s modified Eagle’s medium (DMEM; Life Technologies, Carlsbad, CA, USA) supplemented with 10% heat-inactivated fetal bovine serum (FBS; MP Biomedicals, Tokyo, Japan), 100 c penicillin, and 100 μg/mL streptomycin (Thermo Fisher Scientific, Waltham, MA, USA) at 37 °C in a humidified atmosphere containing 5% CO_2_.

### 2.3. Spheroid Culture

The T98G, U87MG, LN-18, and U251MG cells were seeded onto nonadherent, V-bottom, 96-well plates (PrimeSurface 96U, MS-9096V, Sumitomo Bakelite, Tokyo, Japan) in 10% FBS-supplemented DMEM at a density of 300–5000 cells/well and cultured for 3 days at 37 °C in a humidified atmosphere containing 5% CO_2_.

### 2.4. Expansion of Primary Human NK Cells and Lymphocyte-Activated Killer Cells

Highly purified human NK cell expansion was performed as previously described [27]. Briefly, PBMCs were obtained from 16 mL heparinized peripheral blood from a healthy volunteer (a 41-year-old man). The CD3 fraction of the PBMCs was depleted by RosetteSep™ Human CD3 Depletion Cocktail (STEMCELL Technologies, Vancouver, BC, Canada). The CD3-depleted PBMCs were placed for 14 days in a T25 culture flask (Corning, Steuben, NY, USA) containing AIM V medium (Life Technologies) supplemented with 10% autologous plasma, 50 ng/mL recombinant human IL-18 (rhIL-18, Medical & Biological Laboratories, Nagoya, Japan), and 3000 IU/mL rhIL-2 (Novartis, Basel, Switzerland) at 37 °C in a humidified atmosphere containing 5% CO_2_. The AIM V medium containing 3000 IU/mL rhIL-2 was replenished as necessary.

Lymphocyte-activated killer (LAK) cells were obtained from PBMCs cultured in AIM V medium supplemented with 5% autologous plasma and 3000 IU/mL rhIL-2 at 37 °C in a humidified atmosphere containing 5% CO_2_.

### 2.5. CFSE-Based Cytotoxic Assay

NK cells (5 × 10^5^) were suspended with 1 μg/mL carboxyfluorescein diacetate succinimidyl ester (CFSE; Dojindo Laboratories, Kumamoto, Japan) and incubated for 30 min at 37 °C. For the flow cytometry-based apoptosis assay, the spheroids were co-cultured with 5 × 10^4^ CFSE-labeled NK cells for 24 h. After co-culture, the cells were centrifuged and detached by StemPro Accutase Cell Dissociation Reagent (Thermo Fisher Scientific) at 37 °C for 60 min. Then, the cells were stained with allophycocyanin (APC)-conjugated Annexin V Apoptosis Detection Kit according to the manufacturer’s instructions (BioLegend, San Diego, CA, USA). Apoptotic GBM cells were detected by a BD FACSCalibur flow cytometer (BD Biosciences, San Jose, CA, USA) and analyzed using FlowJo version 10 (BD Biosciences). For accurate analysis, the CFSE-positive fraction was gated out to evaluate the GBM cell apoptosis.

For the fluorescent microscopy-based cytotoxic assays, propidium iodide (PI; Dojindo Laboratories) was added to spheroids derived from 300 cells from each GBM cell line after 24-h co-culture with the CFSE-labeled NK cells (1250–2500) and incubated for 15 min. The spheroids were observed under a BZ-X700 all-in-one fluorescence microscope (Keyence, Osaka, Japan). CFSE-labeled NK cells were detected using the green fluorescent protein (GFP) filter (OP-87763, Keyence); PI-stained apoptotic GBM cells were detected using the Texas Red filter (OP-87765, Keyence). To visualize the cells within all spheroids, merged Z-stack images were recorded using the BZ-X700 quick-full-focus function.

### 2.6. Microarray Gene Expression Assay

The gene expression of the monolayer-culture GBM cell lines was investigated using the Affymetrix Human Genome U133 Plus 2.0 Array (National Center for Biotechnology Information Gene Expression Omnibus [NCBI GEO] database [37], accession no. GSE23806 and GSE42474 accessed on 19 August 2021). Signal values and detection calls were generated using Affymetrix Microarray Suite 5.0 (MAS5). For each probe set on each array, the MAS5 algorithm yields a detection call: Absent (A), Present (P), or Marginal (M), which indicates whether the specific mRNA is detectable. The detection call in MAS5 is based on a non-parametric statistical test (Wilcoxon signed-rank test) of whether significantly more perfect matches show more hybridization signals than their corresponding mismatches [38].

The total RNA of the spheroids derived from the 3-day culture of 5 × 10^3^ GBM cells, human primary NK cells, and LAK cells was extracted with NucleoSpin RNA (Takara Bio, Shiga, Japan). The gene expression of the RNA samples was analyzed by Riken Genesis (Kawasaki, Japan) using the Clariom™ S array. The microarray data were deposited into the GEO database under the accession numbers GSE182373 for GBM-sp and GSE182374 for NK cells and LAK cells, respectively. All CEL files were analyzed using Transcriptome Analysis Console (TAC 4.1, Thermo Fisher Scientific). Gene expression was analyzed with the gene-level Signal Space Transformation Robust Multi-Chip Analysis (SST-RMA) summarization method [39]. The microarray data were normalized by the robust multiarray average method; a probe set was considered expressed if >50% of samples had Detected Above Background (DABG) values below the DABG threshold (*p* < 0.05). The expression status was described True (T) or False (F), indicating whether the specific mRNA was detectable or not. In GBM cell lines, we focused on the genes for GSC markers (NOTCH2, STAT3, MYC, CD44, CXCR4, ITGA6, PDGFRA, L1CAM, NES, SOX2, MSI1, NANOG, CDH5, POU5F1, PROM1, FUT4), ECM markers (COL6A1, FN1, LTBP1, COL1A1, MMP16, SNED1, CDH1, LUM, CFTR, COL4A6, LAMA1, SUSD5), chemokines (CCL1, CCL2, CCL3, CCL3L3, CCL4L2, CCL5, CCL7, CCL8, CCL11, CCL13, CCL14, CCL16, CCL17, CCL18, CCL19, CCL20, CCL21, CCL22, CCL23, CCL24, CCL25, CCL26, CCL27, CCL28, CXCL1, CXCL2, CXCL3, CXCL5, CXCL6, CXCL8, CXCL9, CXCL10, CXCL11, CXCL12, CXCL13, CXCL14, CXCL16, CXCL17, XCL1, XCL2, CX3CL1), chemokine receptors (CCR1, CCR2, CCR3, CCR4, CCR5, CCR6, CCR7, CCR8, CCR9, CCR10, CX3CR1, CXCR1, CXCR2, CXCR3, CXCR4, CXCR5, CXCR6, XCR1), NK cell activation receptor ligands (CD70, CFP, CLEC2B, ITGB2, MICA, NCR3LG1, NID1/PDGFD, TNFSF4, TNFSF9; ligand of CD27, NCR1, KLRF1, ICAM1, KLRK1, NCR3, NCR2, OX40L, CD137, respectively), and NK cell inhibitory receptor ligands (CD274, CDH1/CDH2/CDH4, CEACAM1/HMGB1/LGALS9/PTDSS1, COL17A1, PVR, HLA-E; ligand of PD-1, KLRG1, TIM3, LAIR1, TIGIT, CD96, KLRC1, respectively). In NK and LAK cells, we focused on the genes for NK cell activation receptors (CD244, NCR1, NCR2, NCR3, CD226, ICAM1, TNFRSF4, TNFRSF9, KLRC2, KLRC4, KIR2DS4, KLRF1, CD27), NK cell inhibitory receptors (LAG3, LILRB1, CTLA4, CD33, PDCD1, SIGLEC9, HAVCR2, LAIR1, TIGIT, KIR2DL1, CD96, KLRC1), immune activators (CSF2, IL7, IL15, TNF, IFNG, IL12A, IL1A, IL1B), immune suppressors (IL13, IL23A, TGFBR2, TGFB1, TGFBR3, IL10, CD22), cytotoxicity (TNFSF10, GZMK, GZMA, PRF1, FASLG, GZMB, GZMM, GZMH), chemokines (CCL1, CCL2, CCL3, CCL3L3, CCL4L2, CCL5, CCL7, CCL8, CCL11, CCL13, CCL14, CCL16, CCL17, CCL18, CCL19, CCL20, CCL21, CCL22, CCL23, CCL24, CCL25, CCL26, CCL27, CCL28, CXCL1, CXCL2, CXCL3, CXCL5, CXCL6, CXCL8, CXCL9, CXCL10, CXCL11, CXCL12, CXCL13, CXCL14, CXCL16, CXCL17, XCL1, XCL2, CX3CL1), and chemokine receptors (CCR1, CCR2, CCR3, CCR4, CCR5, CCR6, CCR7, CCR8, CCR9, CCR10, CX3CR1, CXCR1, CXCR2, CXCR3, CXCR4, CXCR5, CXCR6, XCR1). All of these genes are summarized in Supplementary Table S1a,b.

### 2.7. Statistical Analysis

The statistical analysis was performed using Prism8 (GraphPad Software, San Diego, CA, USA). Values are shown as the means ± standard deviation (SD). The statistical significance of differences was determined using one-way analysis of variance (ANOVA) followed by Tukey’s test, and accepted at *p* < 0.05. The OS was compared using a two-sided log-rank test. Survival in each group was estimated using Kaplan–Meier methodology, including medians (95% CI [confidence intervals]) and OS rates. The statistical significance of differences was determined using Tukey’s honestly significant difference (HSD).

## 3. Results

### 3.1. Cancer Stem Cell Marker, ECM Marker, and Chemokine/Chemokine Receptor Expression in Human Glioma

**Figure 1:**
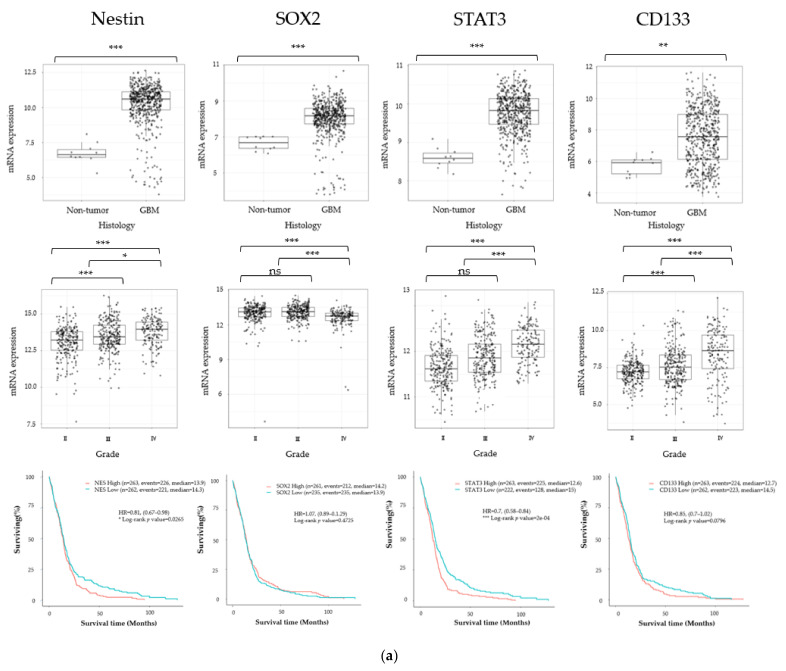

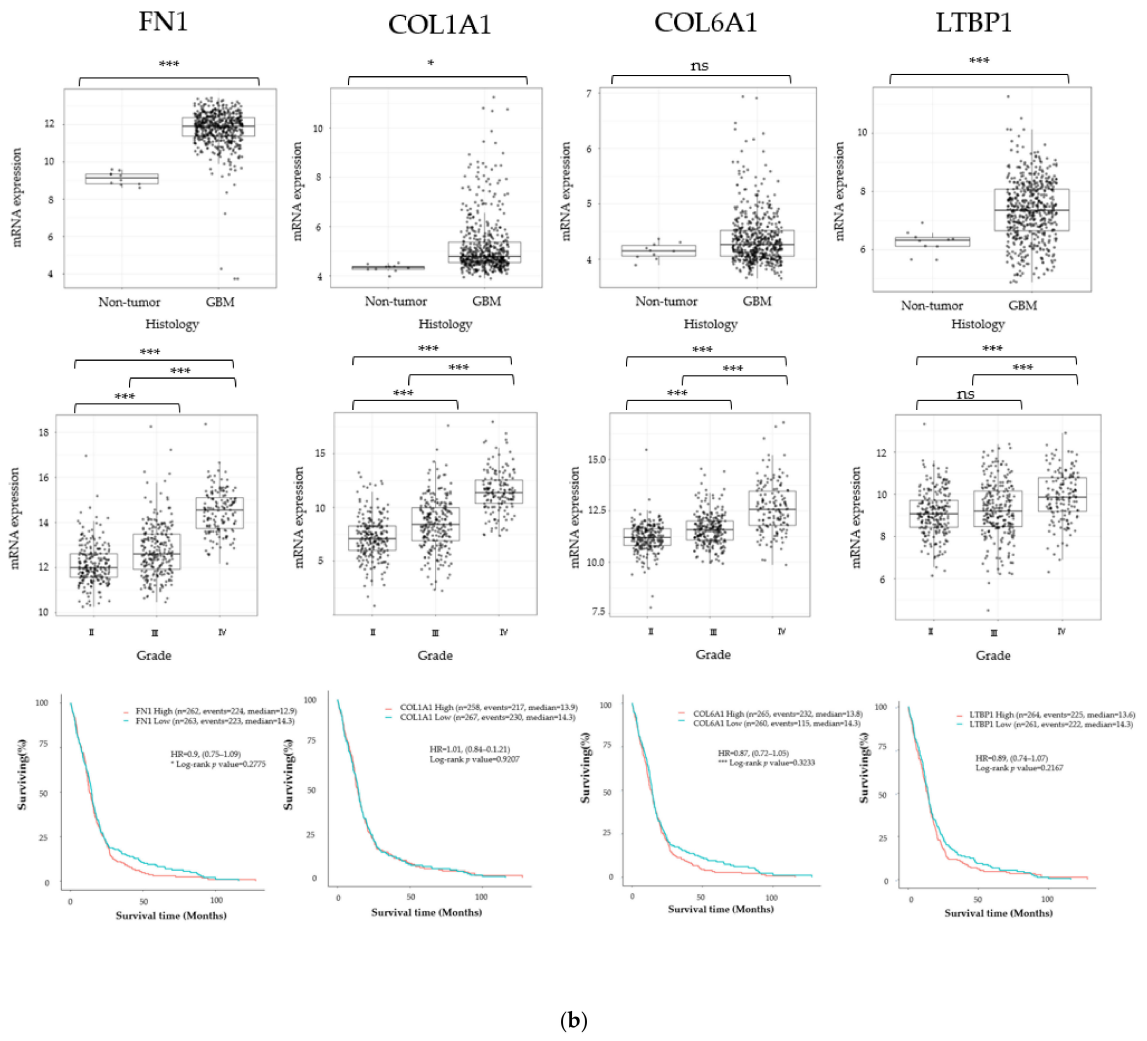

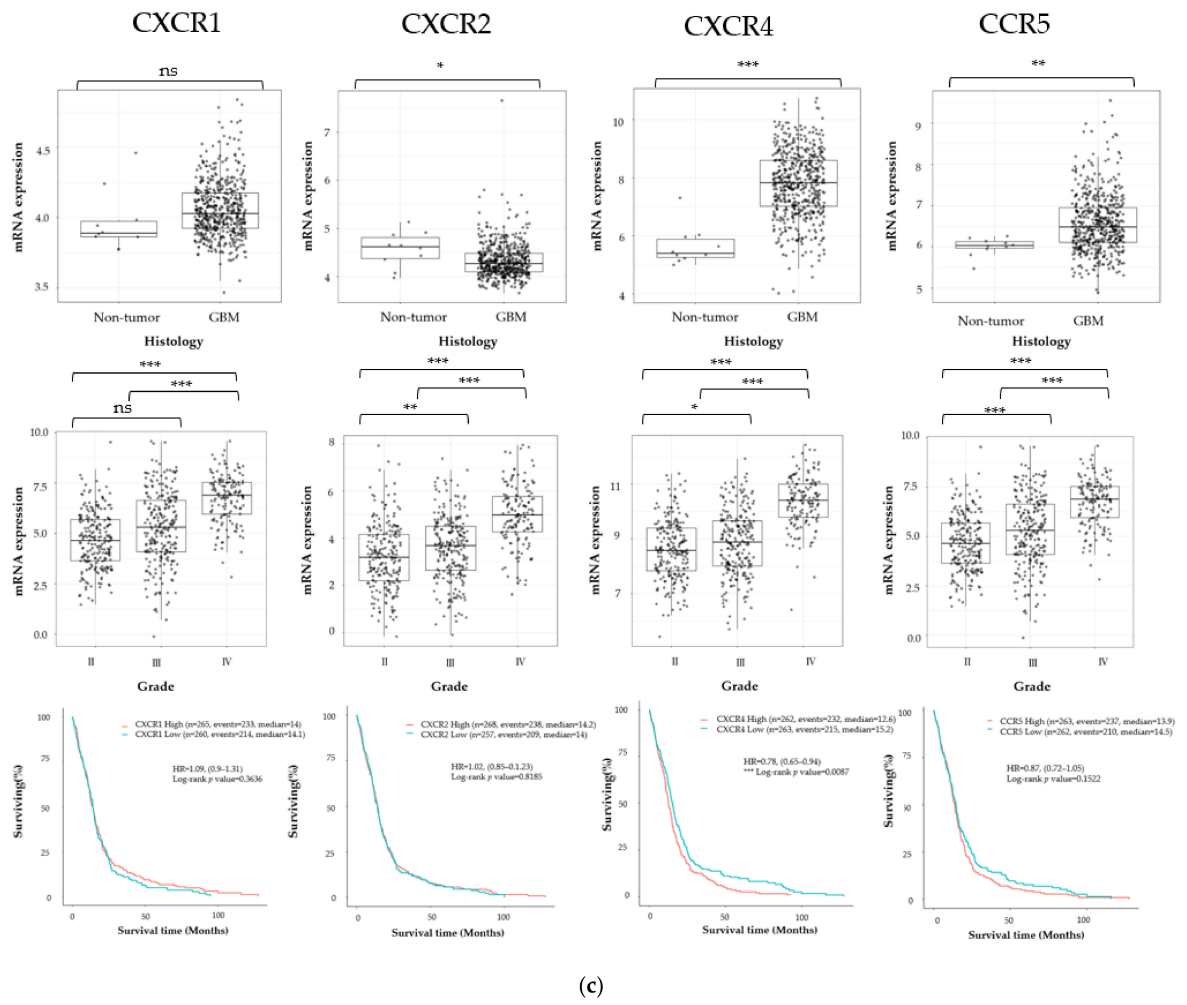

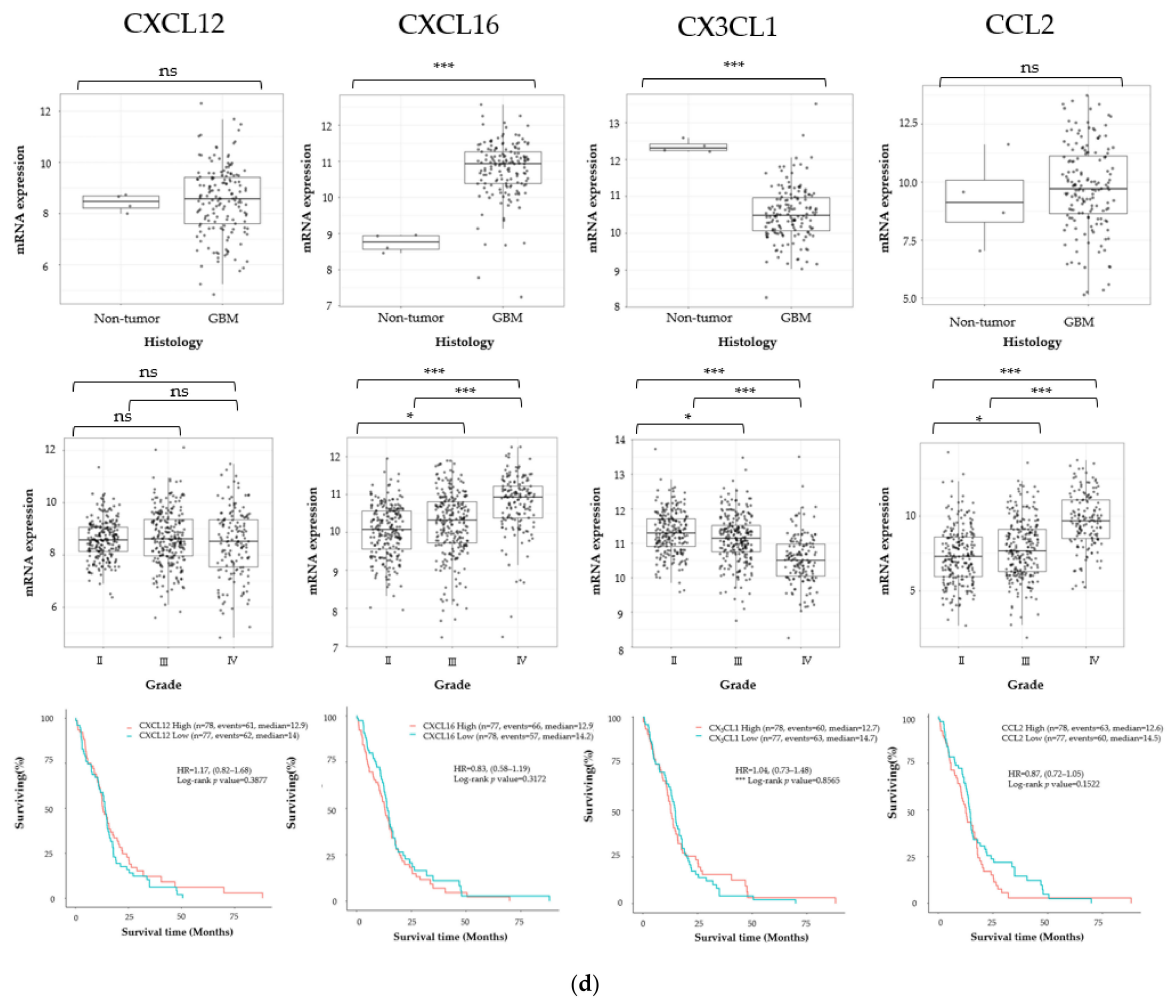
Gene expression of glioma stem cell (GSC) markers (**a**), extracellular matrix (ECM) markers (**b**), chemokine receptors (**c**), and chemokines (**d**) in glioblastoma (GBM). Top: Gene expression in GBM tissue and healthy brain tissue in The Cancer Genome Atlas (TCGA) data set. Middle: Expression of the same genes according to World Health Organization (WHO) grade. The statistical significance of differences was determined using Tukey’s honestly significant difference (HSD). *p < 0.05, **p < 0.01, ***p < 0.001. Bottom: Kaplan–Meier curves based on the expression of each gene. The overall survival (OS) was compared between treatment groups using a two-sided log-rank test; statistical significance of differences was accepted at *p* < 0.05. ns: not significant.

To detect the expression patterns of the representative stem cell markers (Nestin, SOX2, STAT3, CD133), ECM markers (FN1, COL1A1, COL6A1, LTBP1), chemokines (CXCL12, CXCL16, CX3CL1, CCL2), and chemokine receptors (CXCR1, CXCR2, CXCR4, CCR5) in gliomas, we examined the RNA sequencing data of gliomas from the GlioVis data portal [36]. Nestin, SOX2, STAT3, and CD133 were significantly expressed in GBM tissue compared with normal brain tissue in TCGA database. Compared to WHO grade II and grade III glioma, GBM had significantly higher expression of Nestin, STAT3, and CD133. Kaplan–Meier curves following log-rank testing showed that Nestin and STAT3 overexpression predicted significantly poorer OS in TCGA database (Figure 1a). Among the ECM markers, FN1, COL1A1, and LTBP1 were significantly expressed in GBM tissue compared with normal brain tissue. FN1, COL1A1, COL6A1, and LTBP1 were significantly expressed in GBM compared with WHO grade II and III glioma. The overexpression of each gene was not correlated to poor OS prediction in TCGA database (Figure 1b). We also investigated the chemokine receptor and chemokine expression patterns, and found significantly higher CXCR4 and CCR5 expression in GBM than in normal brain tissue. In TCGA database, GBM tissue had higher CXCR1, CXCR2, CXCR4, and CCR5 expression compared with WHO grade II and III glioma. However, only CXCR4 overexpression was significantly related with poor OS in the same database (Figure 1c). GBM tissue had significantly higher CXCL16 expression than normal brain tissue. TCGA database chemokine analysis showed that CXCL16 and CCL2 were significantly expressed in GBM tissue compared with WHO grade II and grade III glioma. However, the representative chemokines in glioma (CXCL12, CXCL16, CX3CL1, CCL2) were not significantly correlated with poor OS in the same database (Figure 1d).

### 3.2. Spheroid Formation and Comprehensive Gene Expression Analysis of 2D Adherent Cells vs. 3D Spheroids Derived from GBM Cell Lines

We examined the expression patterns of the representative GSC markers (NOTCH2, STAT3, MYC, CD44, CXCR4, ITGA6, PDGFRA, L1CAM, NES, SOX2, MSI1, NANOG, CDH5, POU5F1, PROM1, FUT4), ECM markers (COL6A1, FN1, LTBP1, COL1A1, MMP16, SNED1, CDH1, LUM, CFTR, COL4A6, LAMA1, SUSD5), chemokines (CCL1, CCL2, CCL3, CCL3L3, CCL4L2, CCL5, CCL7, CCL8, CCL11, CCL13, CCL14, CCL16, CCL17, CCL18, CCL19, CCL20, CCL21, CCL22, CCL23, CCL24, CCL25, CCL26, CCL27, CCL28, CXCL1, CXCL2, CXCL3, CXCL5, CXCL6, CXCL8, CXCL9, CXCL10, CXCL11, CXCL12, CXCL13, CXCL14, CXCL16, CXCL17, XCL1, XCL2, CX3CL1), chemokine receptors (CCR1, CCR2, CCR3, CCR4, CCR5, CCR6, CCR7, CCR8, CCR9, CCR10, CX3CR1, CXCR1, CXCR2, CXCR3, CXCR4, CXCR5, CXCR6, XCR1), NK cell activation receptor ligands (CD70, CFP, CLEC2B, ITGB2, MICA, NCR3LG1, NID1/PDGFD, TNFSF4, TNFSF9; ligand of CD27, NCR1, KLRF1, ICAM1, KLRK1, NCR3, NCR2, OX40L, CD137, respectively), and NK cell inhibitory receptor ligands (CD274, CDH1/CDH2/CDH4, CEACAM1/HMGB1/LGALS9/PTDSS1, COL17A1, PVR, HLA-E; ligand of PD-1, KLRG1, TIM3, LAIR1, TIGIT, CD96, KLRC1, respectively).

**Figure 2:**
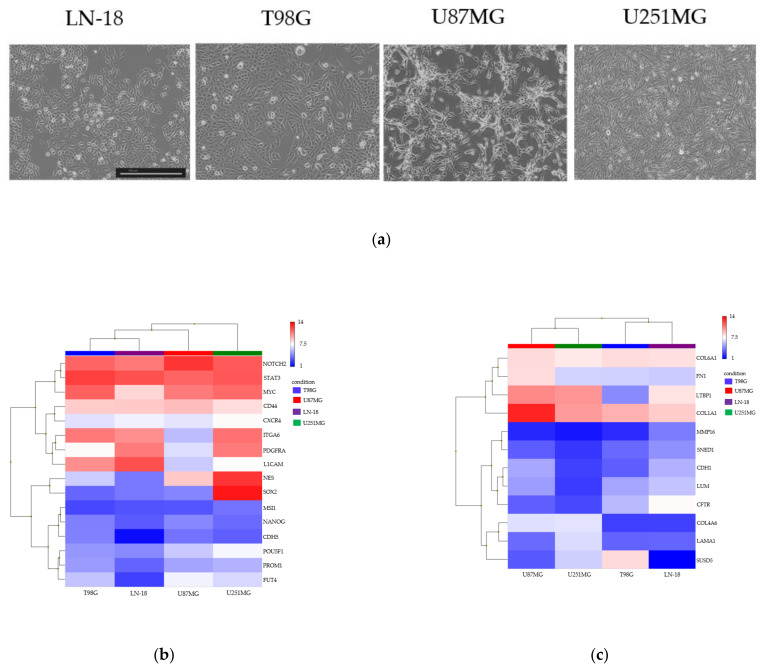

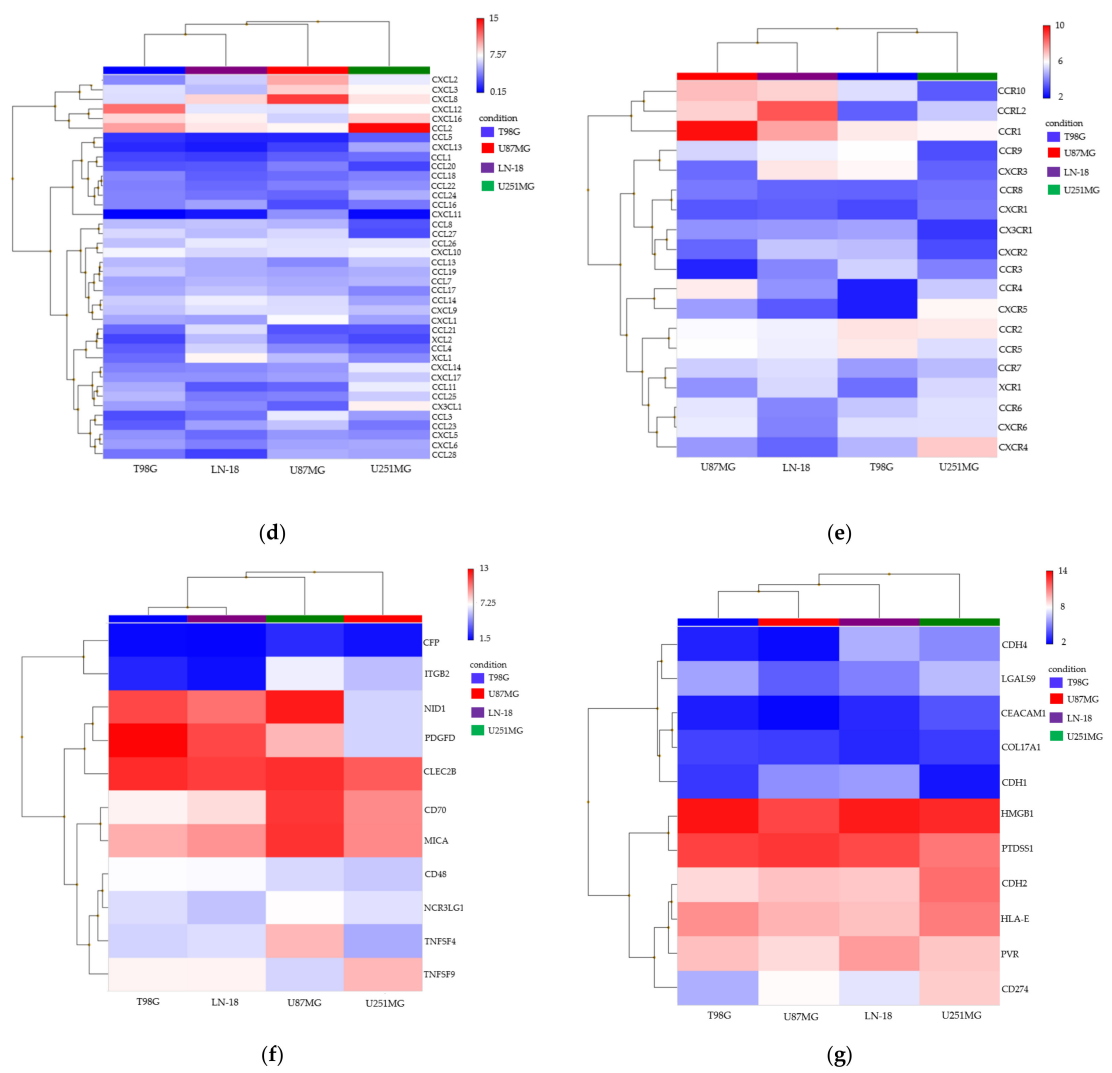
Hierarchical clustering of gene expression in monolayer-culture LN-18, T98G, U87MG, and U251MG GBM cell lines. (**a**) Morphology of each GBM cell line in monolayer-culture conditions; scale bar = 500 μm. (**b**–**g**) The heatmaps of the transcriptome-wide Clariom™ S array of gene expression related to GSC markers (**b**), ECM markers (**c**), chemokines (**d**), chemokine receptors (**e**), natural killer (NK) cell activation receptor ligands (**f**), and NK cell inhibitory receptor ligands (**g**).

In the monolayer-culture GBM cell lines (Figure 2a), the GSC markers STAT3, NOTCH2, PDGFRA, ITGA6, and CD44 were significantly expressed in all cell lines (Figure 2b). Among the ECM markers, COL1A1, COL6A1, and FN1 were significantly expressed in all cell lines (Figure 2c). Among the chemokines, CCL2 and CXCL8 were significantly expressed in all cell lines (Figure 2d). No chemokine receptor was consistently expressed in the cell lines (Figure 2e). Among the NK cell activation receptor and inhibitory receptor ligands, CD70, CLEC2B, MICA, NID1, CDH2, HMGB1, PTDSS1, and PVR were significantly expressed in all cell lines (Figure 2f,g). The respective MAS5 signal/detection call *p*-values are presented in Supplementary Table S2.

**Figure 3:**
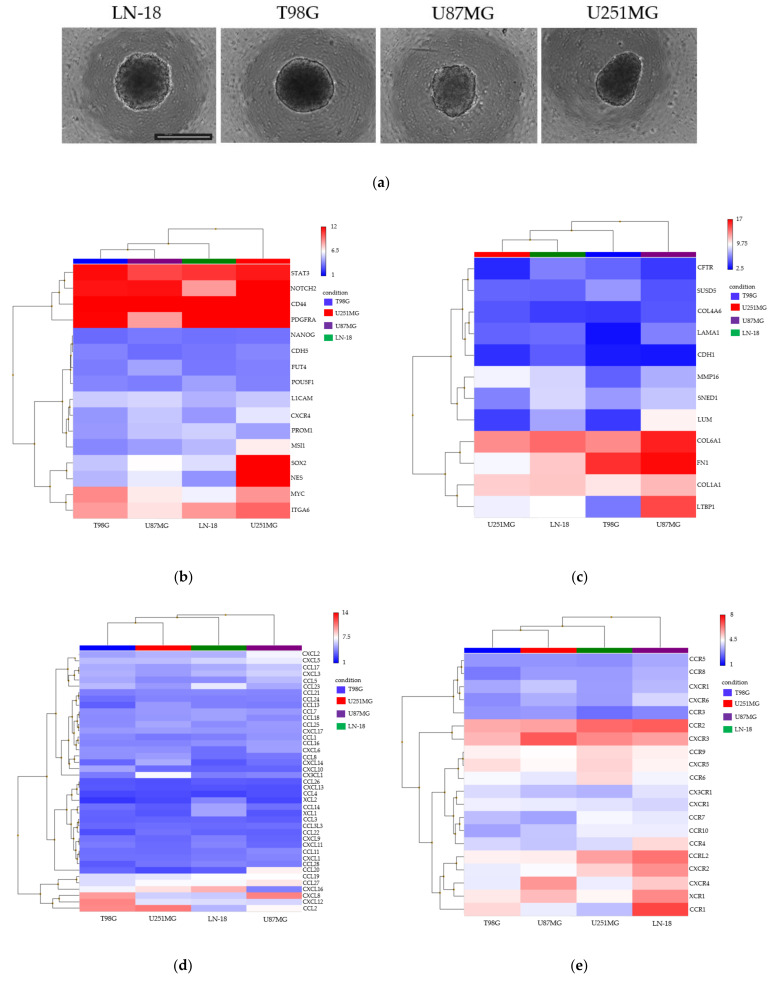

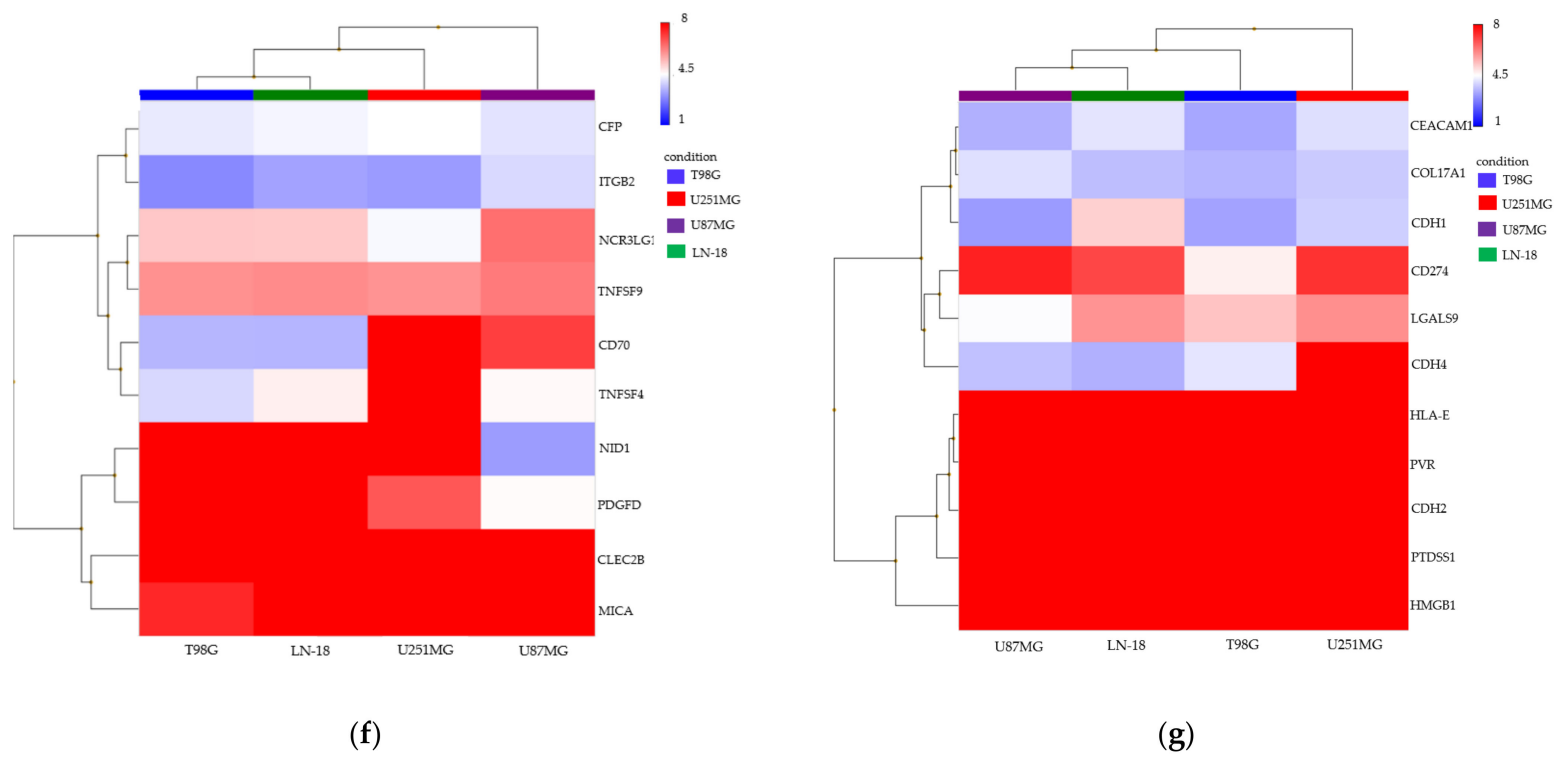
Hierarchical clustering of gene expression in spheroids derived from LN-18, T98G, U87MG, and U251MG GBM cell lines. (**a**) Morphology of each GBM cell line (LN-18, T98G, U87MG, and U251MG) in spheroid culture conditions; scale bar = 500 μm. (**b**–**g**) The heatmaps of the transcriptome-wide Clariom™ S array of gene expression related to GSC markers (**b**), ECM markers (**c**), chemokines (**d**), chemokine receptors (**e**), NK cell activation receptor ligands (**f**), and NK cell inhibitory receptor ligands (**g**).

In the GBM-sp (Figure 3a), the GSC markers STAT3, SOX2, PROM1, POU5F1, PDGFRA, NOTCH2, MYC, MSI1, L1CAM, ITGA6, CXCR4, and CD44 were significantly expressed in all cell lines (Figure 3b). Among the ECM markers, COL1A1, COL4A6, COL6A1, FN1, LTBP1, LUM, MMP16, and SNED1 were significantly expressed in all cell lines (Figure 3c). Among the chemokines, CCL2, CCL7, CCL8, CCL11, CCL13, CCL16, CCL17, CCL19, CCL23, CCL24, CCL25, CCL27, CXCL5, CXCL8, CXCL12, CXCL16, and CXCL17 were significantly expressed in all cell lines (Figure 3d). Among the chemokine receptors, CCR2, CCR7, CCRL2, CXCR2, CXCR3, CXCR4, CXCR5, and XCR1 were significantly expressed in all cell lines (Figure 3e). Among the NK cell activation receptor and inhibitory receptor ligands, CFP, CLEC2B, MICA, NCR3LG1, TNFSF9, CDH274, CDH2, HMGB1, PTDSS1, and PVR were expressed significantly in all cell lines (Figure 3f,g). The respective DABG values are presented in Supplementary Table S2.

**Figure 4:**
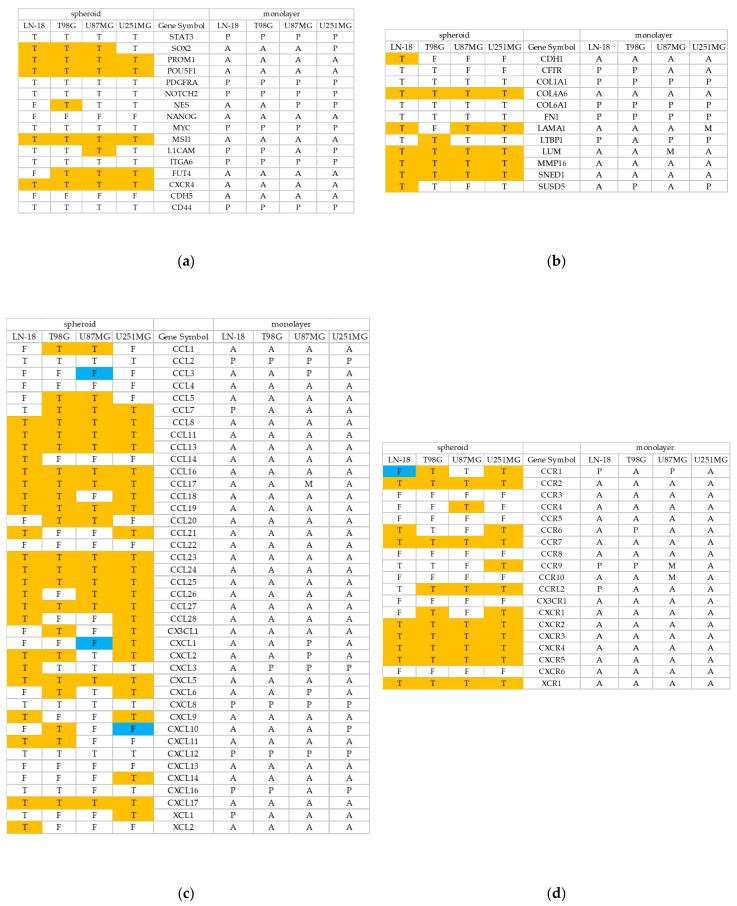

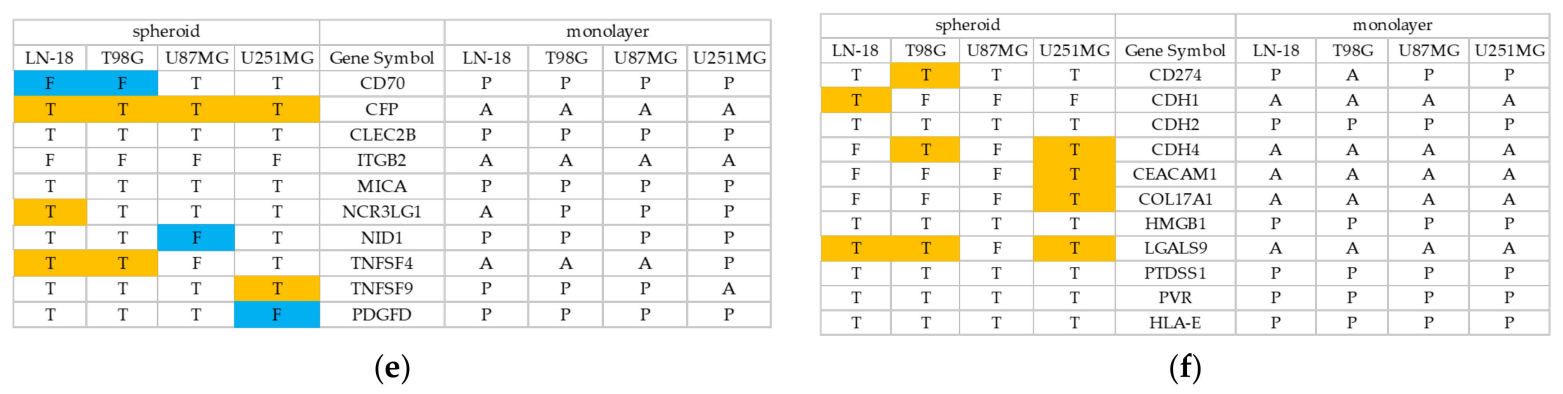
Differential gene expression between monolayer-culture cells and spheroids derived from GBM cell lines. We analyzed gene expression, GSC markers (**a**), ECM markers (**b**), chemokines (**c**), chemokine receptors (**d**), NK cell activation receptor ligands (**e**), and NK cell inhibitory receptor ligands (**f**) in the monolayer culture using Transcriptome Analysis Console (TAC). For each probe set on each array, the Microarray Suite 5.0 (MAS5) algorithm yields a detection call: Absent (A), Present (P), or Marginal (M), which indicates whether the specific mRNA is detectable. The detection call in MAS5 is based on a non-parametric statistical test (Wilcoxon signed-rank test) of whether significantly more perfect matches show more hybridization signals than their corresponding mismatches. We also analyzed gene expression using Detected Above Background (DABG) values. The microarray data were normalized by the robust multiarray average method; a probe set was considered expressed if >50% samples had DABG values below the DABG threshold (*p* < 0.05). The expression status was described as True (T) or False (F), which indicates whether the specific mRNA was detectable. The significantly differentially expressed genes were determined by expression analysis performed using ANOVA. The orange cells indirectly indicate upregulation, where the expression status changed from Absent, which was analyzed by detection calling, to True, which was analyzed by DABG values; the blue cells indirectly indicate downregulation, where the expression status changed from Present to False.

We also analyzed and summarized the differential gene expression between the monolayer cultures and GBM-sp. Gene expression was defined as upregulated when it changed from Absent, which was judged by using detection calling in the monolayer cultures, to True, which was analyzed by the DABG value in the spheroids. Likewise, gene expression was considered downregulated when it changed from Present in the monolayer cultures to False in the spheroids. The GSC markers PROM1, POU5F1, MSI1, and CXCR4 were upregulated in all cell lines (Figure 4a), as were the ECM markers COL4A6, LUM, MMP16, and SNED1 (Figure 4b). The chemokines CCL8, CCL11, CCL13, CCL16, CCL17, CCL19, CCL23, CCL24, CCL25, CCL27, CXCL5, and CXCL17 were upregulated in all cell lines (Figure 4c), as were the chemokine receptors CCR2, CCR7, CXCR2, CXCR3, CXCR4, CXCR5, and XCR1 (Figure 4d). A few NK cell activation receptor ligands were upregulated: CFP in all cell lines, NCR3LG1 in LN-18 cells, TNFSF4 in T98G cells and LN-18 cells, and TNFSF9 in U251MG cells, while some were downregulated: CD70 in LN-18 cells and T98G cells, and NID1 in U87MG cells (Figure 4e). Several NK cell inhibitory receptor ligands were upregulated: CD274 in T98G cells; CDH1 in LN-18 cells; CDH4 in T98G cells and U251MG cells; CEACAM1 in U251MG cells; COL17A1 in U251MG cells; and LGALS9 in T98G, LN-18, and U251MG cells; no genes were downregulated (Figure 4f).

### 3.3. Comprehensive Gene Expression Analysis of the Expanded NK Cells as Compared to Conventional LAK Cells

We reported novel culture systems of human primary NK cells, which were sufficient for attaining high-purity (>98%) expanded (>440-fold) CD3^−^/CD56^+^ peripheral blood-derived NK cells. These were designated GiNK, which exhibited high NK activity and low Treg frequency compared with LAK cells, and the NK cells expressed some NK cell activation receptors and NK cell inhibitory receptors [27]. 

**Figure 5:**
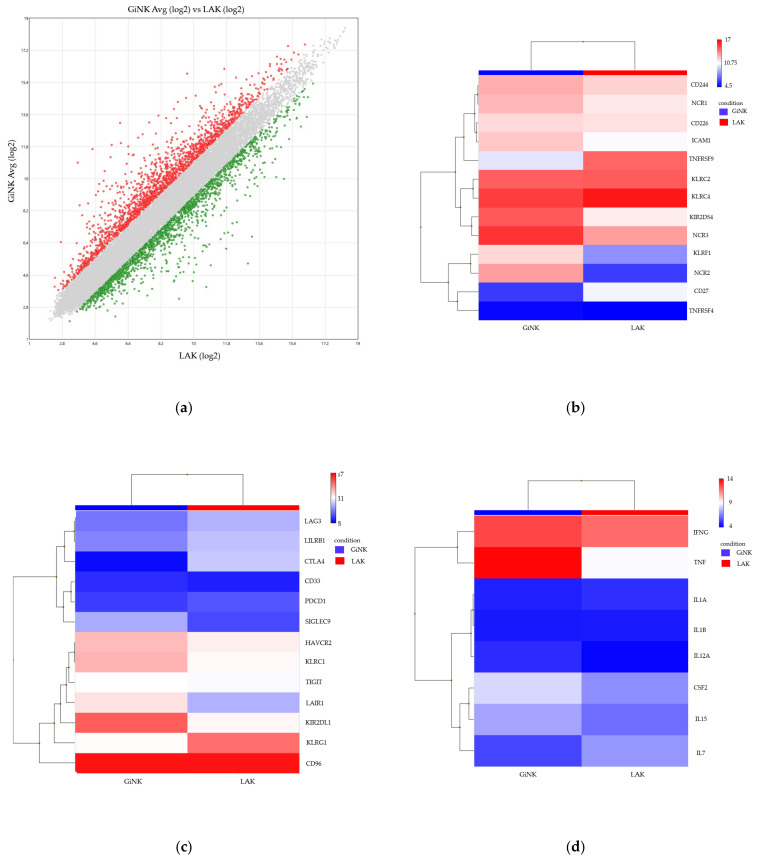

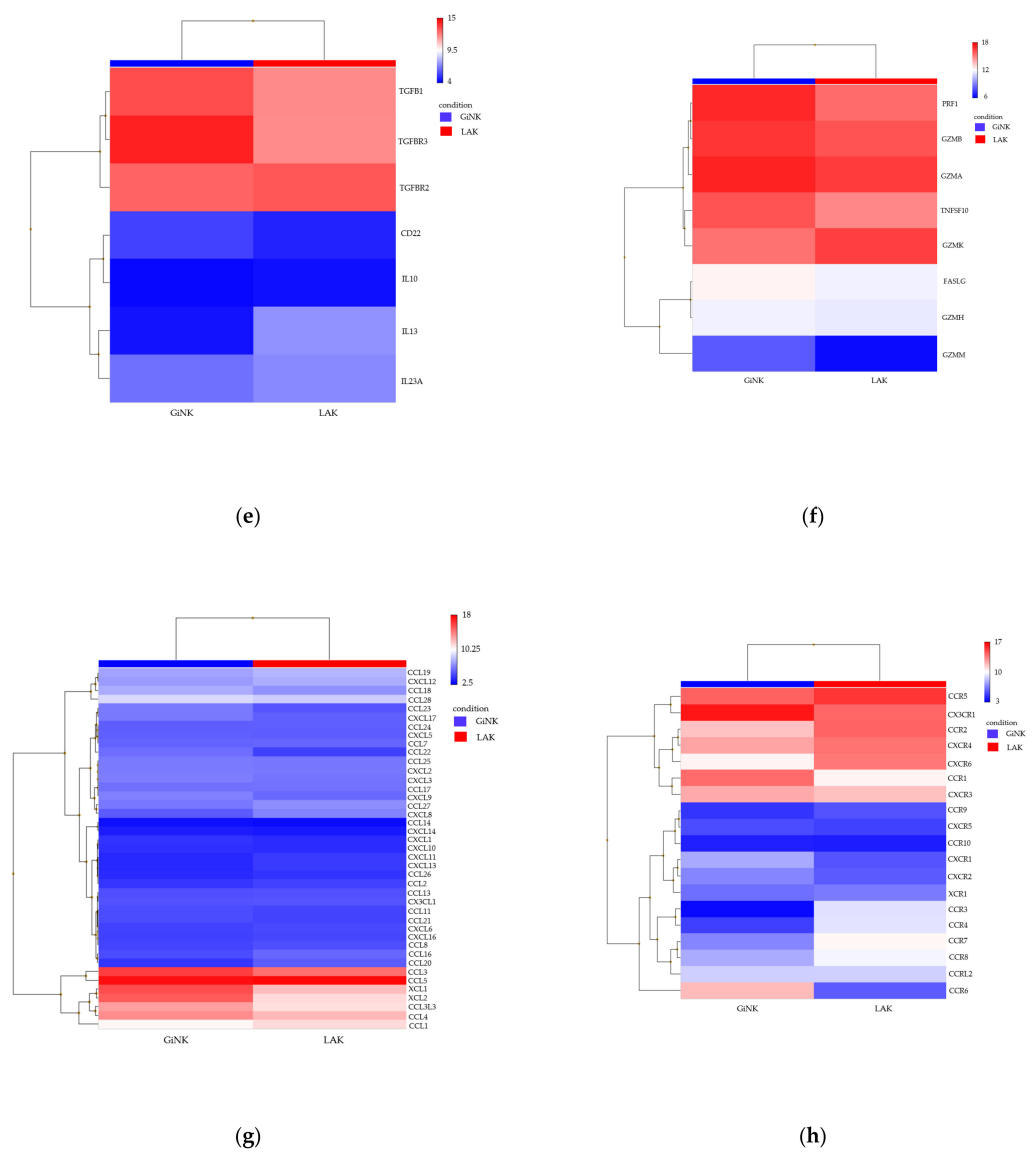
Scatter plot and heatmaps of transcriptome-wide Clariom™ S array of genuine induced NK cells (GiNK) and lymphocyte-activated killer (LAK) cells. (**a**) The scatter plot represents the gene expression of the NK cells and LAK cells (red plot, NK cells; green plot, LAK cells). (**b**–**h**) Heatmaps represent gene expression related to NK cell activation receptors (**b**), NK cell inhibitory receptors (**c**), immune activation (**d**), immune suppression (**e**), cytotoxicity (**f**), chemokines (**g**), and chemokine receptors (**h**).

To identify the differentially expressed genes in the NK cells compared with LAK cells, we profiled gene expression using the Clariom™ S Array GeneChip (Affymetrix) and filtered the differentially expressed genes with ANOVA using TAC 4.1 (Figure 5a). We focused on seven groups of genes related to NK cells: NK cell activation receptors (CD244, NCR1, NCR2, NCR3, CD226, ICAM1, TNFRSF4, TNFRSF9, KLRC2, KLRC4, KIR2DS4, KLRF1, CD27), NK cell inhibitory receptors (LAG3, LILRB1, CTLA4, CD33, PDCD1, SIGLEC9, HAVCR2, LAIR1, TIGIT, KIR2DL1, CD96, KLRC1), immune activators (CSF2, IL7, IL15, TNF, IFNG, IL12A, IL1A, IL1B), immune suppressors (IL13, IL23A, TGFBR2, TGFB1, TGFBR3, IL10, CD22), cytotoxicity (TNFSF10, GZMK, GZMA, PRF1, FASLG, GZMB, GZMM, GZMH), chemokines (CCL1, CCL2, CCL3, CCL3L3, CCL4L2, CCL5, CCL7, CCL8, CCL11, CCL13, CCL14, CCL16, CCL17, CCL18, CCL19, CCL20, CCL21, CCL22, CCL23, CCL24, CCL25, CCL26, CCL27, CCL28, CXCL1, CXCL2, CXCL3, CXCL5, CXCL6, CXCL8, CXCL9, CXCL10, CXCL11, CXCL12, CXCL13, CXCL14, CXCL16, CXCL17, XCL1, XCL2, CX3CL1), and chemokine receptors (CCR1, CCR2, CCR3, CCR4, CCR5, CCR6, CCR7, CCR8, CCR9, CCR10, CX3CR1, CXCR1, CXCR2, CXCR3, CXCR4, CXCR5, CXCR6, XCR1). Among the NK cell activation receptors, ICAM1, KIR2DS5, KLRF1, NCR1, NCR2, and NCR3 expression was upregulated (fold change > 2; fold change = 2.76, 12.45, 13.14, 2.28, 145.04, and 5.94, respectively) (Figure 5b). Among the NK inhibitory receptors, SIGLEC9 expression was upregulated (fold change = 4.97) (Figure 5c). Among the immune activation genes, CSF2, IL15, and TNF were upregulated (fold change = 2.73, 2.13, and 32.37, respectively) (Figure 5d). Among the immune suppression genes, TGFB1 and TGFBR3 were upregulated (fold change = 2.49 and 4.93, respectively) (Figure 5e). The expression of the cytotoxicity-related GZMM, PRF1, and TNFSF10 was upregulated (fold change = 3.58, 3.02, and 2.37, respectively) (Figure 5f). Among the chemokine genes, CCL3, CCL3L3, CCL4, XCL1, and XCL2 were upregulated (fold change = 2.74, 3.76, 2.49, 10.22, and 14.48, respectively) (Figure 5g). Among the chemokine receptor genes, CCR1, CCR6, CXCR1, CXCR2, and CX3CR1 were upregulated (fold change = 13.44, 84.43, 5.06, 2.26, and 4.79, respectively) (Figure 5h).

### 3.4. Cytotoxicity Assay

**Figure 6:**
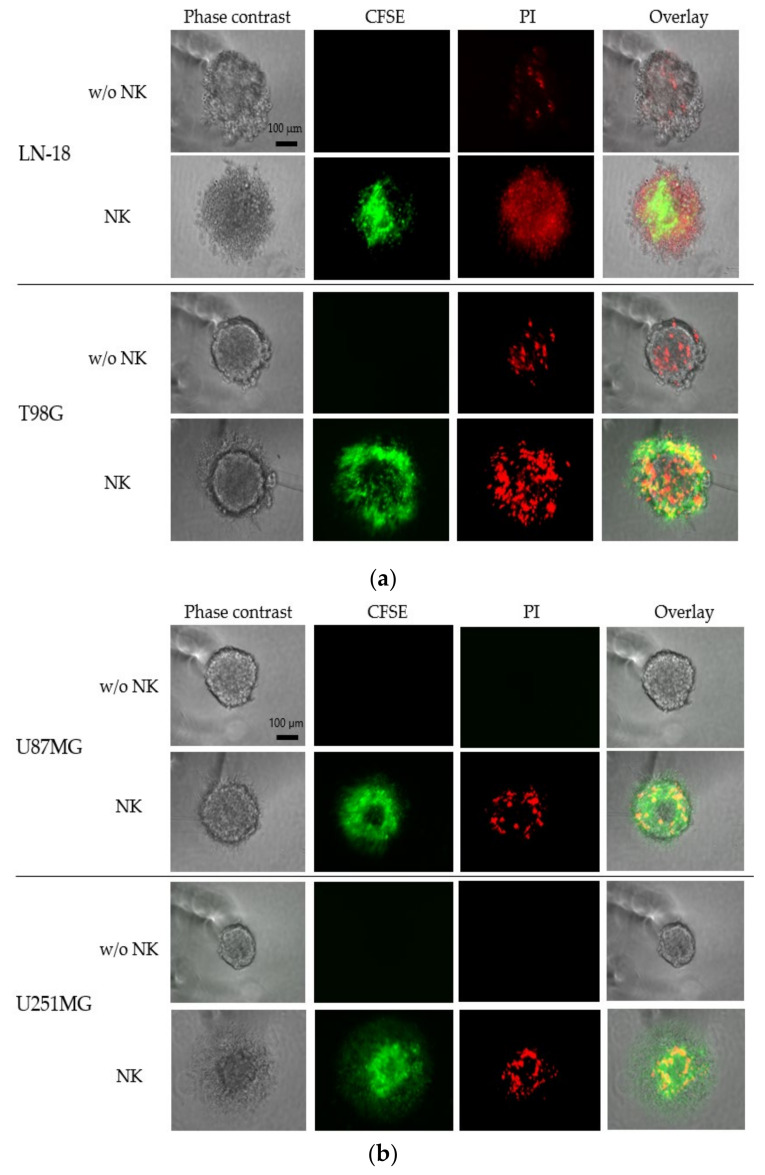
Fluorescent microscopy evaluation of cytotoxicity of the expanded human NK cells against GBM cell-derived spheroids (GBM-sp). The spheroids were co-cultured with carboxyfluorescein diacetate succinimidyl ester (CFSE)-labeled NK cells (green) for 24 h. After incubation, propidium iodide (PI, red) was added to the spheroids and incubated for 15 min. Left panels depict the phase-contrast images of the spheroids derived from LN-18, T98G (**a**), U87MG, and U251MG cells (**b**); scale bar = 100 μm; the second and third row panels depict the fluorescent microscopic images for detecting CFSE and PI. The images on the right depict the overlay view of the CFSE and PI fluorescent microscopic images under phase-contrast imaging.

We performed fluorescent microscopic analysis to investigate the dynamics and apoptosis induction of NK cells against GBM-sp induced from 300 cells. The co-culture conditions were: 1250 CFSE-labeled NK cells were added to LN-18-sp, T98G-sp, and U87MG-sp; 2500 CFSE-labeled NK cells were added to U251MG-sp. After 24 h, the LN-18-sp and T98G-sp included a small population of PI-positive dead cells in the absence of NK cells; the U87MG-sp and U251MG-sp did not contain dead cells. The NK cells induced cell death in all GBM-sp tested. In the T98G-sp and U87MG-sp, NK cells accumulated and infiltrated around the surface of the spheroids, where PI staining was positive in overlay view. In the LN-18-sp and U251MG-sp, the NK cells appeared to infiltrate and accumulate in the spheroids. These data indicate that the NK cells induced cell death in the GBM-sp (Figure 6a,b).

**Figure 7:**
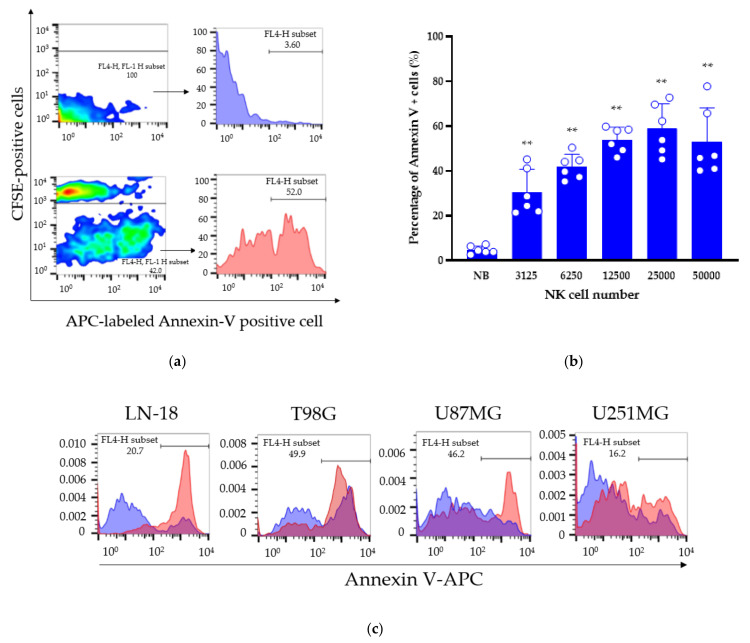

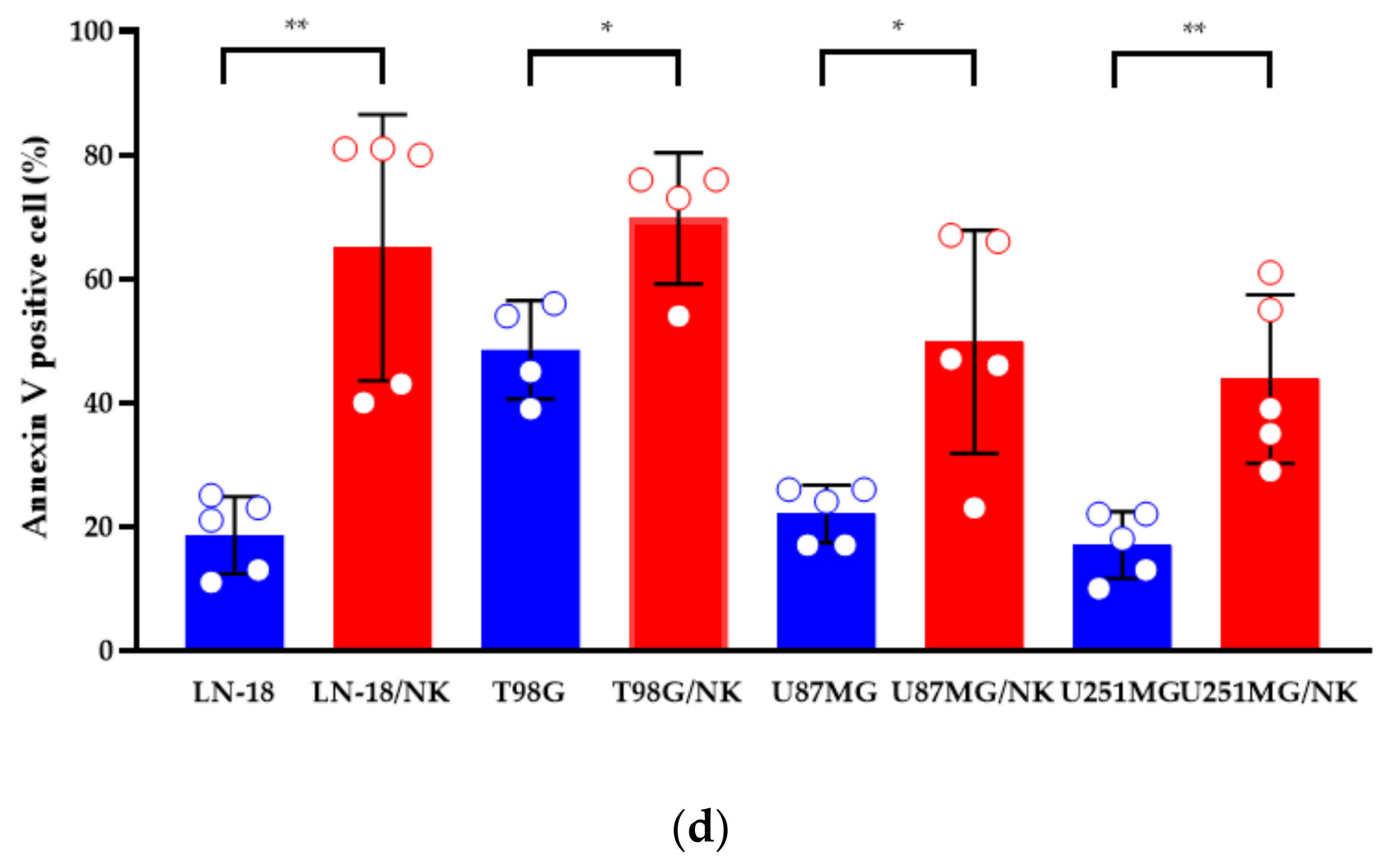
The expanded NK cells induced GBM-sp apoptosis. (**a**) Representative flow cytometric figures in the LN-18-sp induced from 3000 cells co-cultured with 12,500 CFSE-labeled NK cells. Dot plots depict the fluorescent intensity of the CFSE and annexin V–APC (allophycocyanin) fractions (left). Histograms represent annexin V-positive cells gated by CFSE-negative fractions (right). (Top) Spheroids only, (bottom) spheroids co-cultured with NK cells. (**b**) Percentage of annexin V-positive cells in the LN-18-sp induced from 3000 cells according to the number of co-cultured NK cells. (**c**) Histograms depict annexin V-positive cells gated by the CFSE-negative fraction. Blue curve depicts 3-day culture of GBM-sp only; red curve depicts spheroids induced from 3000 cells co-cultured with 50,000 CFSE-labeled NK cells. (**d**) The percentage of annexin V-positive cells. Blue columns depict GBM-sp; red columns depict GBM-sp co-cultured with CFSE-labeled NK cells. Data represent the mean ± SD of at least two independent experiments, where n = 4–5 per group. Significant differences were determined by one-way ANOVA followed by Tukey’s test; *p < 0.01, **p < 0.05.

To confirm the above findings, we evaluated the apoptosis induction effect of the NK cells in the GBM-sp by flow cytometry. The experimental condition was: Spheroids induced from 3000 cells were co-cultured with CFSE-labeled NK cells for 24 h and stained with annexin V–APC. We analyzed annexin V-positive apoptotic cells in the GBM-sp by gating out CFSE-positive fractions. We found that 3125, 6250, 12,500, 25,000, and 50,000 NK cells induced 30.5 ± 10.3%, 41.9 ± 5.6%, 53.9 ± 5.7%, 58.8 ± 11.2%, and 52.9 ± 15.3% annexin V-positive cells, respectively, in the LN-18-sp. The NK cells significantly induced LN-18-sp apoptosis in a cell number-dependent manner up to co-culture with 25,000 NK cells (Figure 7a,b). We investigated the NK cell-mediated apoptosis induction effect against other GBM-sp, and there were 65.0 ± 21.5%, 70.0 ± 10.1%, 49.8 ± 18.0%, 43.8 ± 13.6% annexin V-positive cells in the LN-18-sp, T98G-sp, U87MG-sp, and U251MG-sp, respectively (Figure 7d). In all GBM-sp tested, the NK cells induced apoptosis significantly (Figure 7c,d).

## 4. Discussion

To the best of our knowledge, this is the first report to demonstrate the effectiveness of activated and expanded human primary NK cells in a 3D spheroid model derived from GBM cell lines. The 3D spheroid model was first reported by Hong et al. and they established spheres derived from GBM cell lines in serum-containing or serum-free medium [34]. The spheroids we established were derived from 300 GBM cells, where the cell numbers were smaller compared with the 1 × 10^4^ cells described in the method of Hong et al., and represented exclusively high reproducibility. Hong et al. also demonstrated that LN229 and U251u cells expressed multiple stem cell markers such as Nestin, Sox2, Musashi-1, and CD44, and the spheroids expressed higher levels of these stem cell markers than the monolayer cells. They suggested that the higher stem cell marker expression indicated higher migration and colony-formation potential [34]. Mariam et al. reported that spheroids derived from T98G cells exhibited both invasive and proliferative capabilities [31].

In the present study, expanded and activated human NK cells infiltrated around the spheroids, and flow cytometry-based apoptosis detection clearly showed that the NK cells induced cell death in the spheroids via apoptosis. He et al. reported that U251 cell-derived spheroids were resistant to NK cell-mediated cytotoxicity [40]. Their study differed from our study because they used resting NK cells. Further, we evaluated NK cell-mediated cytotoxicity against additional GBM cell line-derived spheroids.

Previously, we reported that our established NK cells exhibited more expression of NK cell activation receptors than LAK cells. Our expanded primary NK cells demonstrated a high proportion (99.5%) of CD3^−^/CD56^+^ cells without T cells [27]. Considering that the critical adverse effect of allogenic NK immunotherapy in humans is graft-versus-host disease caused by alloreactive T cells, or passenger lymphocyte syndrome caused by donor-derived B cells, the high purification of NK cells plays an important role in avoiding adverse effects in clinical settings [41,42]. In this regard, our allogenic primary NK cells have an advantage for clinical use.

We demonstrated the characteristics of the GBM-sp with microarray. Compared with monolayer-culture GBM cell lines, cancer stem cell (CSC) markers such as SOX2, PROM1, POU5F1, MSI1, FUT4, and CXCR4 were upregulated in the spheroids. Also termed tumor-initiating cells, CSCs are a small subset of cells within malignant tumors that are capable of initiating and driving tumor growth [43,44,45]. CSCs are found in GBM as the so-called GSC [46]. GSC not only have the potential for self-renewal, malignant proliferation, differentiation, and tumorigenicity [47,48,49], but also interact in a multidirectional manner with different tumor components such as the ECM, the cellular compartment (e.g., cancer-associated fibroblasts and immune cells), and the blood–brain barrier to establish a TME, supporting further malignization and treatment resistance [50]. We also found greater upregulation of ECM markers, such as CDH1, COL4A6, LAMA1, LTBP1, LUM, MMP16, SNED1, and SUSD5 in the spheroids than in the 2D culture model. COL4A6, LUM, MMP16, and SNED1, in particular, were upregulated in all cell lines; the results indicate enhanced cell–cell physical interaction [51]. In vitro 3D models have been studied for maintaining GSC multipotency and 3D interactions [52,53]. In vitro 3D models can be classified into spherical cancer models, organoids, and 3D scaffolds. Weiswald et al. classified the spherical cancer model into four types: tumor spheres, multicellular tumor spheroids (MCTS), organotypic multicellular spheroids, and tissue-derived tumor spheres [54]. The spheroid model in the present study involves MCTS, which are cultured with serum-supplemented medium and no additional growth factors in non-adherent conditions [55]. Spheroids have a layered structure: an external layer composed of proliferative cells, an intermediate layer comprising quiescent cells, and an inner acidic, hypoxic layer comprised of necrotic cells [32]. Longati et al. demonstrated that spheroids show enhanced production and deposition of tumor ECM proteins compared to 2D culture models [33]. Given these findings, spheroid models display an anti-cancer therapeutic resistance profile comparable to that of in vivo tumors. In fact, we found upregulated gene expression of GSC and ECM markers, which indicates that our results are consistent with these findings.

We also analyzed the expression of chemokines and their receptors. The chemokines CCL8, CCL11, CCL13, CCL16, CCl17, CCL19, CCL23, CCL24, CCL25, CCL27, CXCL5, and CXCL17 were upregulated in all cell lines, as were the chemokine receptors CCR2, CCR7, CXCR2, CXCR3, CXCR4, CXCR5, and XCR1. There are few studies on the relationship between chemokine–receptor axes with gliomas. CXCL8 and CXCR2 tumor expression is related to glioma grade, disease-free survival, and OS in GBM [56], and their upregulation in high-grade glioma correlates with resistance to anti-angiogenic therapy [57,58]. Increased CXCL12–CXCR4 expression has been detected in patients with GBM [59], and the axis participates in tumor angiogenesis and promotes VEGF production by glioma [60]. Carvalho Da Fonseca et al. reported that CCL2 secreted by glioma cells promotes tumor growth and the migration of malignant cells [61], and the CCL2–CCR2 axis promotes tumor progression by recruiting suppressive myeloid-derived suppressor cells [62,63]. Considering these facts, our findings indicate that the upregulated gene expression patterns of the chemokines and chemokine receptors in the GBM-sp contribute to the resemblance to the characteristics of in vivo tumors. Our results also demonstrate that, despite CCR2 and CXCR4 expression, which is related to leukocyte recruitment to the tumor site mediated by inflammatory chemokines [64], in the human primary NK cells we established, chemokine gene expression was low both in the GlioVis-analyzed patients with GBM and in the microarray-analyzed spheroids; furthermore, few NK cells infiltrate into the GBM [19,20].

The GBM-sp also exhibited differential expression of NK cell activation receptor and NK cell inhibitory receptor ligands. Among the NK cell activation receptor ligands, CFP (NCR1 ligand) was upregulated in all cell lines, while CD70 (CD27 ligand) in LN-18 and T98G cells and NID1 (NCR2 ligand) in U87MG cells were downregulated. Among the NK cell inhibitory receptor ligands, no gene was downregulated, while CD274 (PD-1 ligand) in T98G cells, CDH1 (KLRG1 ligand) in LN-18 cells, CDH4 (KLRG1 ligand) in T98G and U251MG cells, CEACAM1 (TIM3 ligand) in U251MG cells, COL17A1 (LAIR1 ligand) in U251MG cells, and LGALS9 (TIM3 ligand) in LN-18, T98G, and U251MG cells were upregulated. Considering the cancer-immunity cycle, the immunosuppression caused by activating immune checkpoints in the TME plays an important role in the step to inhibiting the killing of cancer cells [65,66]. The GBM-sp we established, with upregulated gene expression of immune checkpoint ligands, exhibited closer properties to GBM in the patient than to the monolayer-culture cell lines.

Shaim et al. reported the αv integrin–TGF-β axis as a potential therapeutic target in GBM [67]. They analyzed GSC derived from primary tumor samples by mass cytometry, and the GSC expressed normal levels of NK cell activation receptor ligands (ULBP2, ULBP3, VIM) and upregulated levels of NK cell inhibitory receptor ligands (HLA-A, HLA-B, HLA-C, HLA-E, HLA-G, PCNA) compared with non-GSC. Close et al. suggested that GBM-infiltrating NK cells express reduced levels of activation receptors within the TME due to TGF-β-mediated inhibition [68]. They analyzed GSC surface antigens with flow cytometry and detected NK cell activation receptor ligands. Given the upregulated expression of the GSC markers, the upregulated expression of NK cell activation and inhibitory receptor ligands are not consistent with that of previous studies.

In summary, our microarray analysis findings reveal that the 3D spheroid model indicates enriched cell growth, progression pathway, and anti-cancer therapeutic resistance. It resembles the complexity of different healthy and diseased human tissues more closely in comparison to the 2D cell culture model. Given the easy preparation and few ethical problems, the spheroid model is useful for exploring the first step in developing a novel strategy for treating patients with GBM.

The present study has some limitations. First, we utilized GBM cell lines, and not patient tissue-derived cells, for the spheroids. Furthermore, cell lines do not reflect the heterogeneity of patient tumors. They undergo massive clonal selection, and genetic drift, so they bear little resemblance to clinical tumors as compared to patient tissue-derived cells [69,70,71]. Although using some patient-derived GBM-sp will yield more scientific value, the cell line model is the fastest means of obtaining preliminary results on the testing of new anti-tumor therapies in vitro as screening because of the easy manipulation and maintenance, with few ethical problems as compared to patient tissue-derived cells. Second, we used peripheral blood from a healthy volunteer to isolate the NK cells. As there is the possibility of patients having an immune function disorder [72], and alkylating agents such as temozolomide inhibit hematopoietic stem cell proliferation and limit lymphocyte numbers in the periphery [2], it would be challenging to isolate NK cells and use them as immunotherapy agents. It is necessary to investigate whether it is possible to isolate sufficient amounts of NK cells to use in a clinical trial. Third, although we showed the differential expression markers in the 2D/3D models via microarray, some of these markers (e.g., MICA) may undergo different post-transcriptional and post-translational regulation. Furthermore, increased expression of these markers at mRNA level is not always correlated with protein surface overexpression. Therefore, as a first step, the present study was aimed at evaluating the overview of the differential expression markers in 2D/3D GBM models, and we did not investigate them in detail. We think that this limitation could form the basis of a further study as the next step in investigating GBM-sp. Fourth, however, the spheroid model exhibited gene expression properties similar to that of human GBM as compared with the monolayer-culture cell lines. There are several models for investigating immunotherapy against GBM: The orthotopic xenograft models provide a central nervous system microenvironment and preserve the integrity of tumor-initiating cells; human stroma and TME are not similar to those in immune-deficient mice [30]. The glioma organoid model has been widely used in basic research and clinical translational research, but lacks a vascular system, which is involved in tumor cell growth and the anti-tumor effect of immune cells [73,74]. Moreover, these models only resemble the human GBM microenvironment; for the purposes of our study, it was sufficient to demonstrate the possibility of immune cell efficacy against tumor cells. For these reasons, the spheroid model is suitable for basic and translational research due to its easy creation and similar gene expression properties to human GBM. Furthermore, we strongly believe that a human clinical trial would be the most effective further investigation and aim to undertake this in the near future.

## 5. Conclusions

We demonstrated the anti-tumor effect of GiNK against GBM cells using an ex vivo 3D GBM cell-derived spheroid model as a preclinical model. Furthermore, we revealed the molecular characteristics of spheroids derived from GBM cell lines. Our findings could lead to the development of future NK cell-based immunotherapies for GBM.

## Data Availability

The data presented in this study are available on request from the corresponding author.

## Supplementary Materials

The following are available online at https://www.mdpi.com/article/10.3390/cancers13194896/s1, Table S1: Gene symbol list analyzed by microarray in NK cells, LAK cells (a), and GBM spheroid (b)., Table S2: Respective MAS5 signal/detection call p-values in monolayer-cultured GBM cell lines and DABG values in GBM spheroid for each gene; GSC (a), ECM (b), chemokines (c), chemokine receptors (d), NK cell activation receptor ligands (e), and NK cell inhibitory receptor ligands (f).
